# Carboxyhemoglobin Formation in Preterm Infants Is Related to the Subsequent Development of Bronchopulmonary Dysplasia

**DOI:** 10.1155/2015/620921

**Published:** 2015-07-29

**Authors:** Shuko Tokuriki, Takashi Okuno, Genrei Ohta, Yusei Ohshima

**Affiliations:** Department of Pediatrics, Faculty of Medical Sciences, University of Fukui, 23-3 Shimoaizuki, Matsuoka, Eiheiji-cho, Yoshida-gun, Fukui 9101193, Japan

## Abstract

*Objective*. To evaluate the usefulness of carboxyhemoglobin (CO-Hb) levels as a biomarker to predict the development and severity of bronchopulmonary dysplasia (BPD). *Methods*. Twenty-five infants born at <33 wk of gestational age or with a birth weight of <1,500 g were enrolled. CO-Hb levels were measured between postnatal days 5 and 8, 12 and 15, 19 and 22, and 26 and 29. Urinary levels of 8-hydroxydeoxyguanosine (8-OHdG), advanced oxidation protein products, and N*ε*-(hexanoyl) lysine were measured between postnatal days 5 and 8 and 26 and 29. Receiver operating characteristic (ROC) analysis was used to compare the biomarkers' predictive values. *Results*. Compared with infants in the no-or-mild BPD group, infants with moderate-to-severe BPD exhibited higher CO-Hb levels during the early postnatal period and higher 8-OHdG levels between postnatal days 5 and 8. Using ROC analysis to predict the development of moderate-to-severe BPD, the area under the curve (AUC) for CO-Hb levels between postnatal days 5 and 8 was higher than AUCs for the urinary markers. *Conclusions*. CO-Hb levels during the early postnatal period may serve as a practical marker for evaluating oxidative stress and the severity of subsequently developing BPD.

## 1. Introduction

Bronchopulmonary dysplasia (BPD) is the most common chronic lung disease in preterm infants [1]. The pathogenesis of BPD is complex, and it involves a variety of causative factors, including excessive oxygen and barotrauma or volutrauma, which are associated with mechanical ventilation and inflammation. Oxidative insults are an important component of the injury process in such cases, as preterm infants are vulnerable to reactive oxygen species owing to the presence of underdeveloped antioxidant systems [2–4]. Many studies have demonstrated that neonates who develop BPD present with elevated levels of oxidative stress markers, which indicate early oxidative deoxyribonucleic acid (DNA) damage, protein damage, and lipid peroxidation even before BPD develops completely [5–7].

Carbon monoxide (CO) has been widely recognized as an exogenous poison. However, CO may also be endogenously formed in a process involving oxidative stress and proinflammatory cytokines [8, 9]; its production is mediated by heme oxygenase (HO) isoforms, particularly HO-1. HO-1 is the rate-limiting enzyme that catalyzes the degradation of heme to CO, iron, and biliverdin. CO binds to hemoglobin (Hb) with a high affinity and forms carboxyhemoglobin (CO-Hb). CO-Hb levels are higher in a number of inflammatory lung diseases, including chronic obstructive pulmonary disease, bronchial asthma, and acute pneumonia [10, 11]. However, whether CO-Hb levels are associated with the development and severity of BPD in premature infants remains unclear.

The aim of this study was to evaluate the relationship between blood CO-Hb levels of preterm infants and the development and severity of BPD. Moreover, we compared the usefulness of blood CO-Hb with that of different urinary oxidative stress markers, namely, 8-hydroxydeoxyguanosine (8-OHdG), a marker of oxidative DNA damage; advanced oxidation protein products (AOPP), a marker of oxidative protein damage; and N*ε*-(hexanoyl) lysine (HEL), a marker of lipid peroxidation, in the evaluation of oxidative injuries in preterm infants [5, 12, 13].

## 2. Patients and Methods

### 2.1. Patient Selection and the Definition of BPD Severity

Preterm infants born at <33 wk of gestational age or with a birth weight of <1,500 g and who were admitted to the neonatal intensive care unit (NICU) at the University of Fukui Hospital between April 2011 and January 2014 were enrolled in this study. The exclusion criteria were congenital malformations, chromosome abnormalities, metabolic disease, severe intraventricular hemorrhages, congenital hemolytic diseases caused by blood-type incompatible pregnancies, diseases requiring surgical treatment, and a gestational age of 22 wk. The study was approved by the Institutional Ethics Committee at the University of Fukui. Parents provided written consent for their children to participate in the study.

BPD and the severity of BPD were defined using the definitions and criteria from the National Institute of Child Health and Human Development Workshop [14]. Mild BPD was defined as a need for supplemental oxygen (O_2_) for ≥28 days plus breathing room air at a postmenstrual age (PMA) of 36 wk if the infants were born at a gestational age of <32 wk, or at a postnatal age of 56 days if the infants were born at a gestational age of ≥32 wk, or at the time of discharge, whichever was first. Moderate BPD was defined as a need for supplemental O_2_ for ≥28 days and a need for treatment with <30% O_2_ at a PMA of 36 wk if infants were born at a gestational age of <32 wk, or at a postnatal age of 56 days if infants were born at a gestational age of ≥32 wk, or at the time of discharge, whichever was first. Severe BPD was defined as a need for supplemental O_2_ for ≥28 days and a need for treatment with ≥30% O_2_ and/or positive pressure at a PMA of 36 wk if infants were born at a gestational age of <32 wk, or at a postnatal age of 56 days if infants were born at a gestational age of ≥32 wk, or at the time of discharge, whichever was first.

### 2.2. Sample Collection and Measurements of Levels of CO-Hb and Urinary Markers

Blood samples (100 *μ*L) were collected during the early postnatal (when infants were aged between 5 and 8 and 12 and 15 days) and late postnatal periods (when infants were aged between 19 and 22 and 26 and 29 days). Blood gas levels, including the levels of CO-Hb, serum bilirubin, and hematocrit, were immediately measured using an ABL800 FLEX analyzer (Radiometer Medical ApS, Brønshøj, Copenhagen, Denmark). CO-Hb levels were expressed as a percentage of the total Hb. Urine samples were collected when infants were aged between 5 and 8 and 26 and 29 days and were centrifuged and stored at −20°C until required for analysis. Urinary levels of 8-OHdG, AOPP, and HEL were measured in duplicate using enzyme-linked immunosorbent assay kits (Japan Institute for the Control of Aging, Nikken SEIL Co., Ltd., Shizuoka, Japan).

In our NICU, antibiotics are routinely administered to preterm infants with respiratory distress at birth or to those in whom sepsis is suspected, for at least 72 h until a diagnosis of bacterial infection is excluded. When infants develop refractory hypotension despite treatment with fluid loading and catecholamines, hydrocortisone is intravenously administered. If infants remain ventilator-dependent and require high-inspired oxygen concentrations after a postnatal age of 1 wk, hydrocortisone or dexamethasone is administered, as needed. Since systemic steroids may affect the CO-Hb levels, the total dosages of hydrocortisone and dexamethasone administered during the first month were calculated by converting the dosage of dexamethasone into that of hydrocortisone.

### 2.3. Statistical Analysis and Receiver Operating Characteristic Analysis

The Mann-Whitney *U*, Wilcoxon signed-rank, and Fisher exact probability tests were used to analyze the data statistically. The Spearman rank correlation coefficient, *ρ*, was used to evaluate correlations between CO-Hb levels and the urinary oxidative stress markers. The data are presented as medians (ranges). A *p* value of <0.05 denoted a statistically significant difference.

Stepwise logistic regression analysis and receiver operating characteristic (ROC) analysis were performed using SPSS software, version 14.0 (IBM, Armonk, NY, USA). To establish a final regression model, downward stepwise selection was used, starting with a prespecified set of candidate variables. An entry criterion of *p* < 0.05 was used for statistical significance during this process. The area under the curve (AUC) was obtained for each marker by plotting the sensitivity and false-positive rate at all possible cutoff points to enable the prediction of the development of moderate-to-severe BPD.

## 3. Results

**Figure 1:**
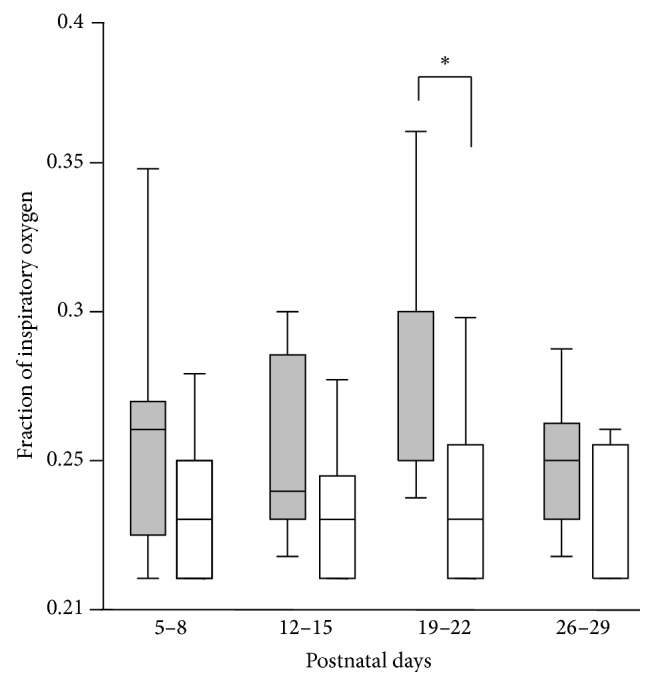
The infants who developed moderate-to-severe bronchopulmonary dysplasia (BPD) required a higher fraction of inspiratory oxygen during the late postnatal period. The fraction of inspiratory oxygen was compared between the no-or-mild BPD group (open bars) and the moderate-to-severe BPD group (closed bars) on the postnatal days indicated. ^*∗*^ *p* < 0.05 for the no-or-mild BPD group versus the moderate-to-severe BPD group on the postnatal days indicated.

During the study period, 168 infants were admitted to our NICU, and 35 had a gestational age of <33 wk or a birth weight <1,500 g. Among them, 3 infants died in the early postnatal period. Six infants were excluded from the study because of severe intraventricular hemorrhage, surgical treatment, and preterm birth at 22 wk. Of the remaining 26 infants, 25 underwent blood and urinary analyses at least once at four different time points (between 5 and 8, 12 and 15, 19 and 22, and 26 and 29 days of age). Twenty-five eligible infants were divided into two groups: the no-or-mild BPD (*n* = 16) and moderate-to-severe BPD (*n* = 9). Compared with the no-or-mild BPD group, the moderate-to-severe BPD group had a younger gestational age (*p* = 0.02), lower birth weight (*p* = 0.01), lower Apgar score at 5 min (*p* = 0.02), and a tendency to require a higher fraction of inspiratory oxygen (Table 1, Figure 1). There were no statistically significant differences between groups with respect to sex, the administration of antenatal steroids, the incidence of chorioamnionitis, septic episodes, surfactant therapy, the duration of mechanical ventilation, the total dosages of hydrocortisone administered, and the number of infants treated with phototherapy (Table 1).

**Figure 2:**
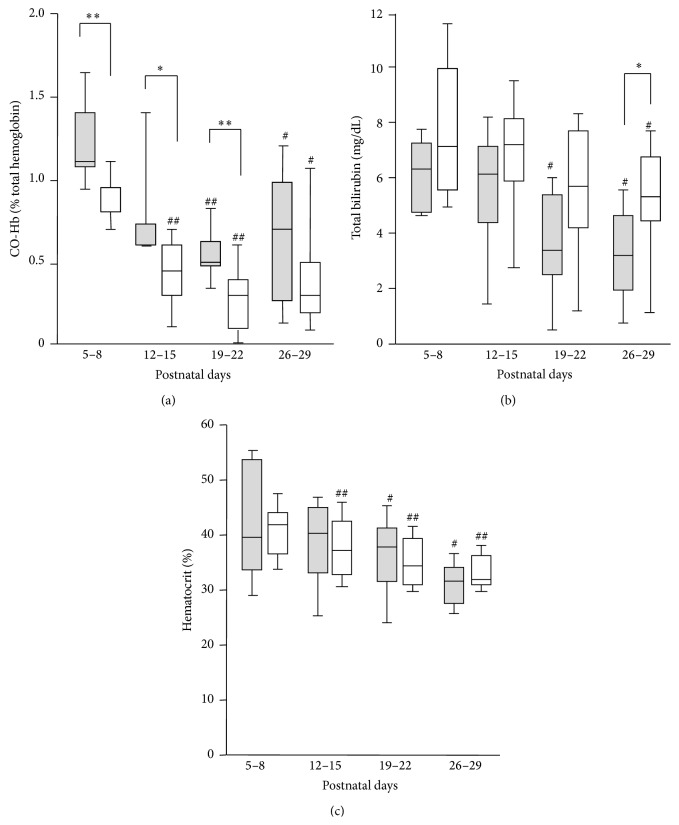
Blood carboxyhemoglobin levels (CO-Hb) but not total bilirubin and hematocrit levels during the early postnatal period correlated with the subsequent development of BPD. The blood levels of CO-Hb (a), bilirubin (b), and hematocrit (c) were compared between the no-or-mild BPD group (open bars) and the moderate-to-severe BPD group (closed bars) on the postnatal days indicated. ^*∗*^ *p* < 0.05 and ^*∗∗*^ *p* < 0.01 for the no-or-mild BPD group versus the moderate-to-severe BPD group on the postnatal days indicated. ^#^ *p* < 0.05 and ^##^ *p* < 0.01 comparison with postnatal days 5–8 of the same group.

CO-Hb levels gradually decreased as infants aged (Figure 2(a)). The moderate-to-severe BPD group exhibited higher levels of CO-Hb compared with the no-or-mild BPD group between postnatal days 5 and 8 (median [range]: 1.1% [0.9–1.8%] versus 0.8% [0.6–1.4%], *p* < 0.01), 12 and 15 (median [range]: 0.6% [0.6–1.8%] versus 0.4% [0.0–0.7%], *p* < 0.05), and 19 and 22 (median [range]: 0.5% [0.3–0.9%] versus 0.3% [0.0–0.6%], *p* < 0.01). Total bilirubin and hematocrit levels also showed a decreasing tendency according to the postnatal ages (Figures 2(b)-2(c)). Although hemolysis and hyperbilirubinemia may affect the CO-Hb levels, there were no significant correlations between the levels of CO-Hb and hematocrit or bilirubin (data not shown).

**Figure 3:**
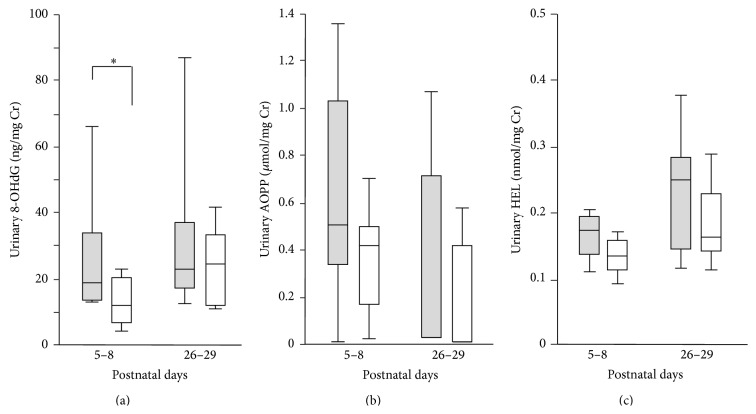
Urinary levels of 8-hydroxydeoxyguanosine (8-OHdG) during the early postnatal period correlated with the subsequent development of BPD, but urinary levels of advanced oxidative protein products (AOPP) and N*ε*-(hexanoyl) lysine (HEL) showed no such correlation. The urinary levels of (a) 8-OHdG, (b) AOPP, and (c) HEL were compared between the no-or-mild BPD (open bars) group and the moderate-to-severe BPD group (closed bars) on the postnatal days indicated. ^*∗*^ *p* < 0.05 for the no-or-mild BPD group versus the moderate-to-severe BPD group on the postnatal days indicated.

Between postnatal days 5 and 8, the urinary 8-OHdG levels in the moderate-to-severe BPD group were significantly higher than those in the no-or-mild BPD group (median [range] creatinine [Cr]: 18.8 ng/mg [13.1–86.6 ng/mg] versus 11.9 ng/mg [3.6–26.6 ng/mg], *p* < 0.05) (Figure 3(a)). The urinary AOPP and HEL levels in the moderate-to-severe BPD group tended to be higher than those in the no-or-mild BPD group between postnatal days 5 and 8, but the differences between groups were not statistically significant (Figures 3(b)-3(c)). There were no significant differences between groups with respect to the urinary levels of 8-OHdG, AOPP, or HEL between postnatal days 26 and 29.

**Figure 4:**
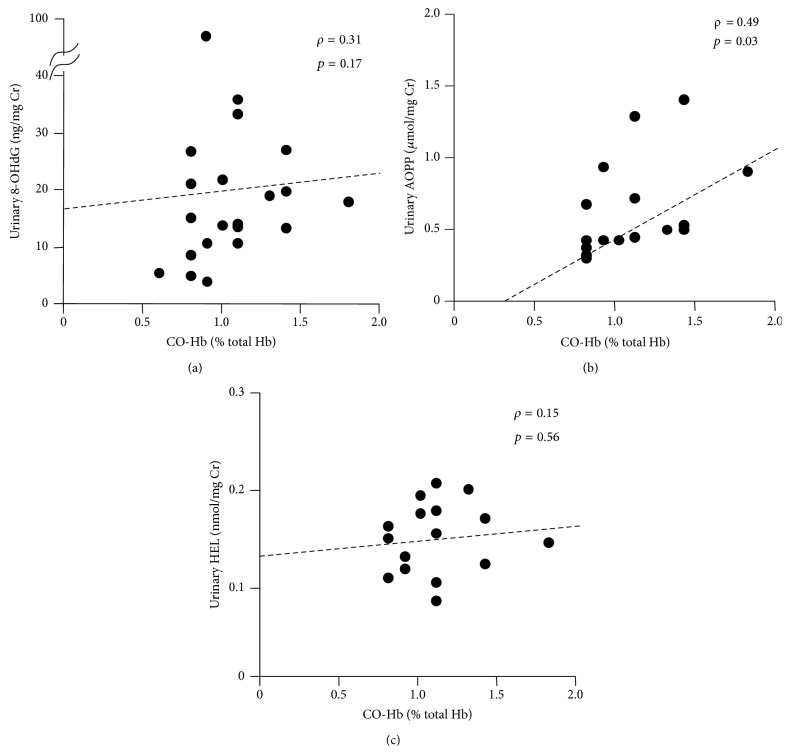
Correlations between the CO-Hb levels and each urinary oxidative stress marker between postnatal days 5 and 8. (a) 8-OHdG, (b) AOPP, and (c) HEL.

CO-Hb levels observed between postnatal days 5 and 8 showed a moderate correlation with the urinary AOPP levels (*ρ* = 0.49, *p* = 0.03) and a weak correlation with urinary 8-OHdG levels (*ρ* = 0.31, *p* = 0.17) on the same postnatal days, but they did not correlate with the urinary HEL levels (Figure 4).

**Figure 5:**
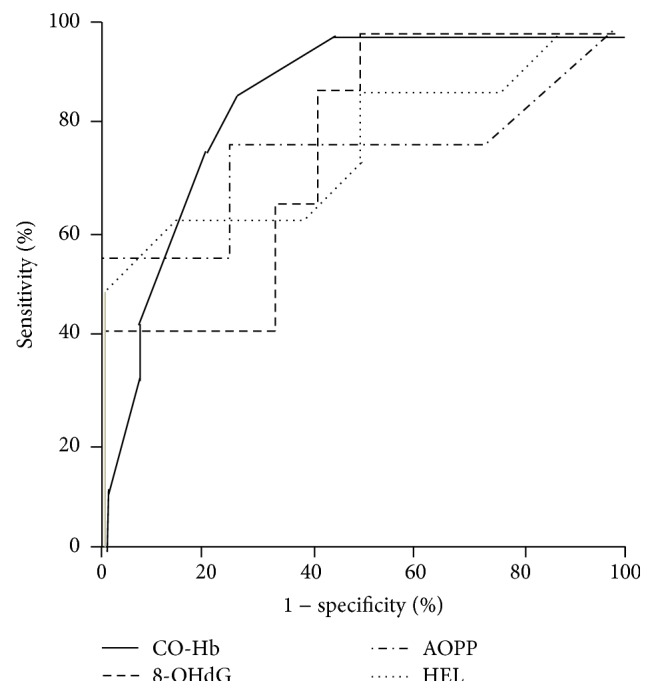
Receiver operating characteristic curves for carboxyhemoglobin and the urinary oxidative stress markers between postnatal days 5 and 8. CO-Hb = carboxyhemoglobin; 8-OHdG = 8-hydroxydeoxyguanosine; AOPP = advanced oxidative protein products; HEL = N*ε*-(hexanoyl) lysine.

To compare the usefulness of CO-Hb levels as a predictive marker for the subsequent development of moderate-to-severe BPD, we performed ROC analysis. The AUC for CO-Hb levels between postnatal days 5 and 8 (0.882, standard error: 0.067, *p* = 0.002) indicated that CO-Hb levels may be a more useful marker than urinary oxidative stress markers for predicting moderate-to-severe BPD (Figure 5, Table 2). With a cutoff value of 1.0%, the sensitivity, specificity, positive predictive value (PPV), and negative predictive value (NPV) of CO-Hb levels were 88.9%, 75.0%, 66.7%, and 92.3%, respectively (Table 2). Compared with the CO-Hb levels, urinary 8-OHdG levels between postnatal days 5 and 8, with a cutoff value of 13.4 ng/mg for Cr, showed a similar sensitivity but a lower specificity, PPV, and NPV.

Finally, to exclude the possibility that CO-Hb levels were a confounding factor of gestational age, birth weight, or neonatal asphyxia in the prediction of severe BPD, we conducted stepwise logistic regression analysis. As shown in Table 3, CO-Hb levels between postnatal days 5 and 8 were identified as the sole predictive risk factor in the final model.

## 4. Discussion

In this study, we demonstrated for the first time that CO-Hb levels gradually decreased as infants aged, and the CO-Hb levels during the early postnatal period are indicative of the severity of BPD that subsequently occurs in preterm infants.

Homolysis is one of the important factors affecting CO-Hb levels [15]. Most endogenous CO is a by-product of red blood cell destruction. Neonatal erythrocytes are more susceptible than adult erythrocytes to oxidative stress [16]. The sudden change in O_2_ concentration at birth, from the low O_2_ environment of the uterus to the high O_2_ concentration outside of the uterus, readily induces HO-1 expression, which catalyzes the cleavage of the heme porphyrin ring and produces CO. Thus, the effects of hemolysis can explain the gradual decline in the CO-Hb, bilirubin, and hematocrit levels in preterm infants as they aged. However, when we compared the no-or-mild BPD group with the moderate-to-severe BPD group at the same postnatal ages, there were no significant differences in the bilirubin and hematocrit levels, which suggest that the higher CO-Hb levels observed in infants who developed moderate-to-severe BPD may be indicative of ongoing oxidative stress insults rather than physiological hemolysis.

Shi et al. [17] observed that plasma CO levels at birth were increased in infants with severe neonatal hypoxic ischemic encephalopathy. If the lower Apgar scores of the moderate-to-severe BPD group were due to neonatal asphyxia, reperfusion-induced oxidative stress following asphyxia may have caused lung injury and compensatory induction of HO-1, resulting in the deterioration of subsequent BPD and the elevation of CO-Hb levels in the moderate-to-severe BPD group during the early postnatal period, respectively [18].

HO-1 has been reported to provide cellular protection against oxidant injury. Fernandez-Gonzalez et al. [19] reported that HO-1 overexpression improved pulmonary inflammation, arterial remodeling, and right ventricular hypertrophy in a murine model of hyperoxia-induced BPD, but it did not prevent alveolar simplification, which is one of the characteristic pathological findings of BPD [20]. They also demonstrated that the protective responses were partially mediated by CO. In our present study, the CO-Hb levels were higher in preterm infants according to the severity of BPD. Since endogenous produced CO binds to hemoglobin promptly and forms CO-Hb, the antioxidative effect may be insufficient to prevent the development of BPD. Therefore, we believe that CO-Hb is a marker reflective of oxidative damage and compensatory induced HO-1.

May et al. reported that end-tidal carbon monoxide (ETCO) levels increased in infants who were born prematurely and developed BPD, and the ETCO levels on postnatal day 14 were predictive of the development of BPD [21, 22]. Considering the half-life of CO-Hb, ETCO levels may represent mainly local and short-term CO production, whereas CO-Hb levels may be associated with relatively long-term integrated CO production [23]. Unlike CO-Hb, however, the measurement of ETCO requires a sophisticated device, and it is difficult to measure ETCO levels in infants who require high frequency oscillatory ventilation or nasal continuous positive airway pressure ventilation.

The administration of corticosteroids reduces ETCO levels [24]. Although there were no differences between the no-or-mild and moderate-to-severe BPD groups in relation to the cumulative dosages of hydrocortisone administered within the first 7 days and the first 28 days of birth, infants who developed severe BPD may have received larger amounts of systemic corticosteroid treatment until their blood was sampled. In this context, even if the CO-Hb levels may be underestimated in the moderate-to-severe BPD group compared to the no-or-mild BPD group, our findings suggested that CO-Hb levels measured during the early postnatal period can be substituted for ETCO measurements and that they may predict the development of BPD.

Joung et al. [5] demonstrated that high urinary 8-OHdG levels on postnatal day 7 are a risk factor for the development of moderate-to-severe BPD, and they concluded that oxidative damage plays a crucial role in the pathogenesis of BPD. In the present study, urinary levels of 8-OHdG, AOPP, and HEL tended to be higher between postnatal days 5 and 8 in infants who developed moderate-to-severe BPD compared with infants in the no-or-mild BPD group. Therefore, the pathogenesis of BPD involves oxidative damage to DNA, proteins, and lipids. However, CO-Hb levels did not necessarily strongly correlate with the levels of urinary oxidative stress markers, implying that, in addition to being indicative of general oxidative stress, they may also be indicative of other factors associated with the exacerbation of BPD, such as ongoing inflammation.

ROC analysis demonstrated that the AUC for CO-Hb levels between postnatal days 5 and 8 was higher than the AUCs for urinary oxidative stress markers between postnatal days 5 and 8. Although urinary sampling is noninvasive, measuring urinary markers is time consuming. Moreover, the data of CO-Hb are readily obtained as a part of routine blood gas analysis. Thus, we believe that CO-Hb levels appear to be more useful than urinary oxidative stress markers in practical settings for estimating general oxidative stress and ongoing inflammation.

There are some limitations of the present study. In addition to hemolysis and asphyxia, several factors such as intrauterine infection and sepsis [25] have been shown to affect CO-Hb levels. Although there was no significant difference in the incidence of chorioamnionitis and septic episodes, further studies are needed to analyze the effects of these factors on the evaluation of oxidative stress using CO-Hb levels.

## 5. Conclusions

In conclusion, our data indicated that CO-Hb levels may provide a readily accessible biomarker of oxidative damage and ongoing inflammation in the immature lungs of infants. CO-Hb levels and urinary oxidative markers can improve our understanding of the role of oxidative stress in the development of BPD.

## Figures and Tables

**Table 1:** Demographic data of the infants in the no-or-mild bronchopulmonary dysplasia (BPD) group and in the moderate-to-severe BPD group.

	No-or-mild BPD (*n* = 16)	Moderate-to-severe BPD (*n* = 9)	*p* value
Gestational age (weeks)	30 (27–32)	27 (26–32)	0.02
Birth weight (g)	1,374 (908–1,982)	1,162 (670–1,434)	0.01
Boys/girls, *n*/*n*	10/6	3/6	0.23
Antenatal steroids, *n*	15	9	>0.99
Chorioamnionitis, *n*	7	4	>0.99
Apgar score (1 min)	7 (2–9)	3 (1–8)	0.06
Apgar score (5 min)	8 (4–10)	5 (2–9)	0.02
Septic episodes, *n*	2	3	0.31
Surfactant therapy, *n*	11	9	0.12
Mechanical ventilation (days)	7 (0–32)	8 (4–46)	0.12
Total dosage of hydrocortisone (mg/kg)	1.0 (0.0–20.0)	2.0 (2.0–13.8)	0.09
Phototherapy (postnatal days 0–4), *n*	15	9	>0.99
Phototherapy (postnatal days 5–8), *n*	4	1	0.62
Phototherapy (postnatal days 12–15), *n*	1	0	>0.99
Phototherapy (postnatal days 19–22), *n*	0	0	
Phototherapy (postnatal days 26–29), *n*	0	0	

Data are expressed as numbers, *n*, or the medians (ranges).

The Mann-Whitney *U* test and Fisher's exact probability test were used for the statistical analyses.

BPD: bronchopulmonary dysplasia.

**Table 2:** Cutoff values for carboxyhemoglobin and the urinary oxidative stress markers for predicting the development of moderate-to-severe bronchopulmonary dysplasia.

Parameter	Cutoff value	Sensitivity	Specificity	PPV	NPV	AUC
CO-Hb	1.0%	88.9%	75.0%	66.7%	92.3%	0.882
Urinary 8-OHdG	13.4 ng/mg Cr	88.9%	58.7%	61.5%	87.5%	0.778
Urinary AOPP	0.43 *μ*mol/mg Cr	77.8%	75.0%	70.0%	81.8%	0.750
Urinary HEL	0.17 nmol/mg Cr	62.5%	87.5%	83.3%	70.0%	0.773

AOPP: advanced oxidation protein products, AUC: area under the curve, CO-Hb: carboxyhemoglobin, Cr: creatinine, HEL: N*ε*-(hexanoyl) lysine, NPV: negative predictive value, 8-OHdG: 8-hydroxydeoxyguanosine, and PPV: positive predictive value.

**Table 3:** Predictors of development of moderate-to-severe bronchopulmonary dysplasia: results of the established model.

Predictors	Univariate analysis			Multivariate analysis		
	Unadjusted OR	95% CI	*p* value	Adjusted OR	95% CI	*p* value
The initial model						
CO-Hb	1166.90	4.00–342463.50	0.015	145.10	0.30–70726.30	0.115
Gestational age	0.56	0.32–0.98	0.041	1.18	0.51–2.75	0.696
Birth weight	1.00	0.99–1.00	0.042	1.00	0.99–1.00	0.487
Apgar score (5 min)	0.59	0.37–0.95	0.029	0.69	0.37–1.30	0.254
The final model						
CO-Hb				**1166.90**	**4.00**–**342463.50**	**0.015**

CO-Hb: carboxyhemoglobin, OR: odds ratio, and CI: confidence interval.
